# Supplementary material for: The masquerade game: marine mimicry adaptation between egg-cowries and octocorals
Source: PeerJ. 2016 Aug 2;4:e2051. doi: 10.7717/peerj.2051 (PMC4975025; doi:10.7717/peerj.2051)

Supplementary information from: Juan A. Sánchez, Angela Fuentes-Pardo, Íde Ní Almhain, Néstor Ardila-Espitia, Jaime Cantera-Kintz & Manu Forero-Shelton "The Masquerade Game: the natural history of marine mimicry adaptation between egg-cowries and octocorals" *PeerJ*

Development of a board game for teaching, playing and outreach

Inspired by the results of the main study, as well as on some elements of the classroom kit from the California Academy of Sciences "Coral Reef: Science and Conservation Game- The fragile coral reef (grades 3-7)" (Teaching and Student Services, 2008), we conceived a board game based on the mimicry adaptation of egg-cowries to coral hosts for teaching some of the underlying processes behind evolution and ecology. We created a simple game by introducing common board game elements such as dice, a square board, and playing cards. Based on observations of the egg-cowries and *Pacifigorgia* spp. (sea fans) at Malpelo Island, we included cards that reflect the events that were observed (see main text) and the probabilities of those events were fine-tuned over several rounds of game development. We included 'impact cards' that added information on natural and catastrophic events thereby defining the life or death of the 'coral game pieces' (Table S1). We also created 'event cards' related to the life cycle of egg-cowries and sea fans (Table S1). The final version of the game presented here summarizes the valuable input received from authors and colleagues who played it multiple times. In brief, this game envisions a biologically relevant driving process that promotes its understanding via the player's exposure to natural processes in ecology and evolution.

The chosen name was 'The Masquerade Game' due to the type of mimicry observed in egg-cowries and its potential ludic meaning concordant with our results. In addition to the cards, we use a 6x6 square board (equally divided into two territories, colonized by two sea fan types), two dice of different color, numerous cowry-like pieces of two different colors (e.g., beans), and two different toy-like pieces to represent the two kinds of predators. Players choose a side (a sea fan population of a specific color) and are given the same amount of randomly chosen colored "egg-cowries" (six) to start with. The game begins with each player placing the cowries on the board and ends when either all sea fan squares (on one side) have been colonized by at least one cowry of their matching color or when only cowries of one color remain.

Since the goal was to develop a game that faithfully represents the natural system, we included several natural events in the dynamics of the game: group migration of the adult egg-cowries for reproduction, hatching of the resulting eggs, mismatched cowries being more susceptible to predators, as well as external factors such as the effects of sea fan mortality (disease and invasive species). Each turn cards assigned to natural events are drawn, and dice specify the location of the events, if required (each die for the x or y position on the board), except for predators, which have a physical presence on the board. The goal of the game is for each player to fill each and all of the squares of their color (Color 1 or color 2) with at least one cowry of the same color. There can

be no more than six cowries in one particular square, following the idea that sea fans are a limited resource and cannot sustain overpopulation.

The element of chance is an important factor, but to make the game more interesting we allowed players some control over certain outcomes, so that strategy could be employed. We did this in various ways. First, we allowed each player to choose which egg-cowry to move on each turn and the direction of its movement. Also, when a reproduction card is drawn, the player can choose which adults move for reproduction if two or more are equidistant from the reproduction site. We also allowed players to choose the predator´s path on the board during their turn. Finally, when overcrowding occurs due to hatching of the egg-cowries, players can choose which new cowries to remove (which results in the removal of mismatched recruits) and where to place offspring within a given region. A kit to play the game is available (figures S1A and table S1). However, it is important to us that the game be easily accessible; for that reason it can be played with six sided dice and a standard 52-card deck and a printed board (Table S2) or even a chess board (Figure S1B) (using part of the board). A summary of the rules is presented in the Table S3 and Card kit at the end.

Remarks. We developed a board game whose main scientific objective was to support the idea that there are conditions for which our hypothesis results in a viable process. Events with high probability were chosen to be those that occur in a regular and almost predictable manner, and are the main drivers of population dynamics including cowry movement, reproductive aggregation, recruitment onto a coral host of the larvae as well as predation. Less probable events are of the disturbance type, which are less frequent and may have catastrophic results on the population and include disease, invasive species as well as a super-predator.

We were actually surprised that playing the game was actually entertaining and included several moments of excitement for people we invited to play. The game lasted an average of a half hour, and the standard distribution of that time was relatively low, with some outliers, especially in the early versions of the game. As expected, the game evolved towards an equilibrium in which either population of egg-cowries colonized their respective sea fan with a few mismatched cowries. This manual simulation based on a game supports the idea that a small number of mismatches due to reproductive aggregations can be maintained over time despite the existence of predators. Two implementations of the game in action can be seen in figures S2 A and B.

Creating a board game based on biologically meaningful rules was not the only motivation for developing the masquerade game. We also wanted to create a game that could go beyond its original inspiration, i.e., it can be played with non-biological subjects and yet be biologically meaningful. We aimed to provide implicit and emergent cognition on natural history and selection as well as the element of chance in nature. Given the difficulty of teaching mainstream biology in some schools (Yates & Marek, 2014), this kind of game

can be an aid to the biology curriculum in the same fashion that computer games and other ludic activities are (Sadler et al., 2013). Our goal is not evaluating its role in formal education, instead, in this context, the board game is similar to a very basic simulation of population dynamics on the one hand, and on the other hand it is an opportunity to intertwine outreach activities in the scientific process, as opposed to it being an afterthought. Inclusion of educational material with a scientific article may contribute to bringing young students closer to the scientific process first hand and is consistent with the current trend in many journals to include sections such as an "Author Summary" for non-specialists.

We believe that an integration of the outreach activities in the scientific process can result in a better integration of the community in the scientific process, an improved understanding of science by the community, and potentially better science since the community gets an opportunity to contribute ideas as well as knowledge. We firmly believe that natural history provides very good examples to promote the outreach of ecology and evolution, particularly when these examples are drawn from the student´s surroundings. Furthermore, a game whose outcomes are organically discovered by students is a form of active learning that could be used as a platform for teaching the scientific method and concepts in evolution, ecology, and conservation, which may support the teaching of mainstream biology in some regions where it competes with other interpretations (Long, 2012).

A game may be as useful as simulations to explore different outcomes of an evolutionary process. Simulations are important tools in that they can explore the parametric space of interest in a short amount of time, but have fundamental limitations: first, since the rules are set from the beginning, a computer simulator cannot question them or come up with new strategies. This intentionality in human players–that so far is difficult to program–can lead to players asking questions about the assumptions, the rules, think of alternative processes and other key aspects of the process under study that a computer would not be able to come up with without an actual understanding of the phenomenon. While designing this game we had to go through multiple iterations of the rules, and this process forced us to think about many possible natural events that can affect the game–and the natural history–in a way that makes it playable and relevant. Gaming can then be thought as a complementary tool to predict and model a natural system; it can be used to explore the large conceptual and parameter space of the natural world and focus on a part of it.

Table S1. Highly and less probable events from the natural history of *Simnia avena* and *Simnialena rufa* egg-cowries and *Pacifigorgia* spp (sea fans). at Malpelo Island (Colombian Pacific, TEP), turned into 52 'event cards' for 'the masquerade game'.

Events/Card Type	Number of cards	Description	Action during game
**Highly Probable events**	**Total of 43**	These are natural history events that occur in a regular and almost predictive manner, which are the main drivers of population dynamics.	These events alter the number or position of cowries on the board, so helping to win the game but also to break down the uniformity that the players seek to win the game. Some cards require the player to throw two dice of different colors. One will represent the position on the x-axis (x-color dice) and y-axis (y-color dive) where the event will happen.
Reproductive aggregation	**9**	This is the major finding of this study, all types of egg-cowries color gather for reproduction and ovoposition into encapsulated eggs on the sea fan surface. As in other ovulids, both males and females exhibit gregarious behavior an gather on a single sea fan colony after copulating with multiple males briefly earlier (Nowlis, 1993).	Throw the dice to determine the position; the closest cowries will congregate in that square.<br>The number of cowries to participate is shown on the card.<br>If a number of cowries are equidistant the player may choose which to move.<br>If all cowries are of the same color the player with this color may keep the card for use in recruitment
Recruitment	**9**	Cowries develop on the encapsulated egg into a	The number of offspring is shown on the card.

		unknown planktonic phase (Bandel, 1973). Afterwards, they settle on coral hosts.	If the player has a reproduction card, (they can play this to ensure all offspring are their color) the player will put cowries on that square and/or any adjacent square.<br>If the number of cowries in any square is more than 6, any additional cowries die; but the order in which the cowries are placed on the squares is chosen by the player playing the card.
Predator	**9**	In the study area, there are two likely predators of cowries: the coral hawkish (*Cirrhitichthys oxycephalus*) and the Malpelo endemic twinspot triplefin *Lepidonectes bimaculatus* (Chasqui Velasco, Gil-Agudelo & Nieto, 2011). Since, these two fishes are common it is assumed that predation on cowries is occasional and in response to camouflage mismatches to due movements or reproductive aggregations.	The player moves the predator (from it´s previous position). The number of squares are shown on the card. The predator cannot move diagonally. In each square that it visits during the movement it will eat any cowries whose color is not matched to the background coral´s color.
Movement	**16**	Here, it is assumed that eventually cowries can move for no particular reason and look for another sea fan host.	Players move one cowry at every turn, and here they can move any of the cowries of their own color, which increases the chances for winning the game; move freely (one square, forward, back sideways or diagonally).

**Less probable events**	**Total of 9**	These are events of the disturbance type, which are less frequent and have catastrophic results on the population.	These movements, as in an intermediate disturbance scenario, will shake the fate of game drastically. Moreover, these cards can delay the player that was close to win and it introduces evenness between players. The player who gets this card must throw two dices of different color to determine the position as above.
Super predator	**2**	The longnose hawkfish (*Oxycirrhites typus*) is a predator that also has a masquerade type background-matching mimicry for the same kind of sea fan (*Pacifigorgia*). It is closely associated to gorgonians and black corals (Béarez, Bujard & Campoverde, 2007). Its sharp and projecting mouth provides it with a lethal tool for picking up prey concealed on sea fans.	This predator eats even background matching cowries.<br>The player chooses a corner of the square and all cowries in squares adjacent to this corner (up to 4 squares) are removed.
Invasive Species	**3**	The snowflake coral *Carijoa riisei* is an invasive octocoral, presumably from the Western Atlantic, which overgrows and kills entirely sea fans at the Tropical Eastern Pacific-TEP (Sánchez & Ballesteros, 2014). This is possible due to an associated sponge	At the chosen position, the invasive species will displace the cowries' coral host. All cowries on this square must move out of it, and the player who got the card decides which of the adjacent squares to place each cowry in.

		on the surface of the coral, which has cytotoxic effects on contact (Calcinai, Bavestrello & Cerrano, 2004).	
Sea fan Disease	**4**	The fungal disease aspergillosis (*Aspegilus sydowi* and *A. flavus*) has been recently detected infecting sefans in the Eastern Tropical Pacific (Barrero-Canosa, Dueñas & Sánchez, 2012). This disease could be related to warming seawater temperatures and land-borne pollution (Sánchez et al., 2014). It is responsible for most of mortality on sea fan hosts in Malpelo and other TEP areas (Sánchez et al., 2012).	All cowries on this square die, so they are removed.

A)

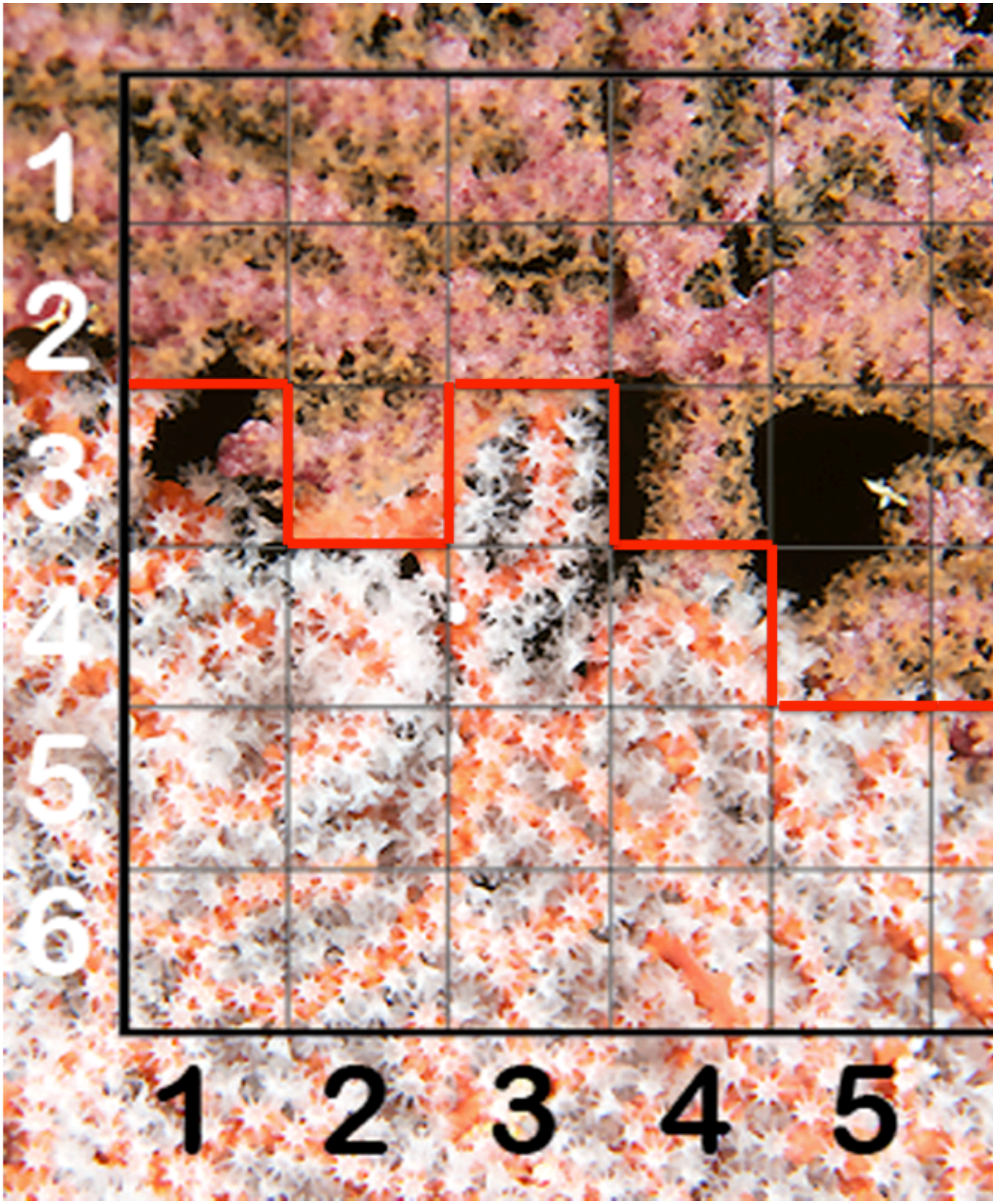

B)

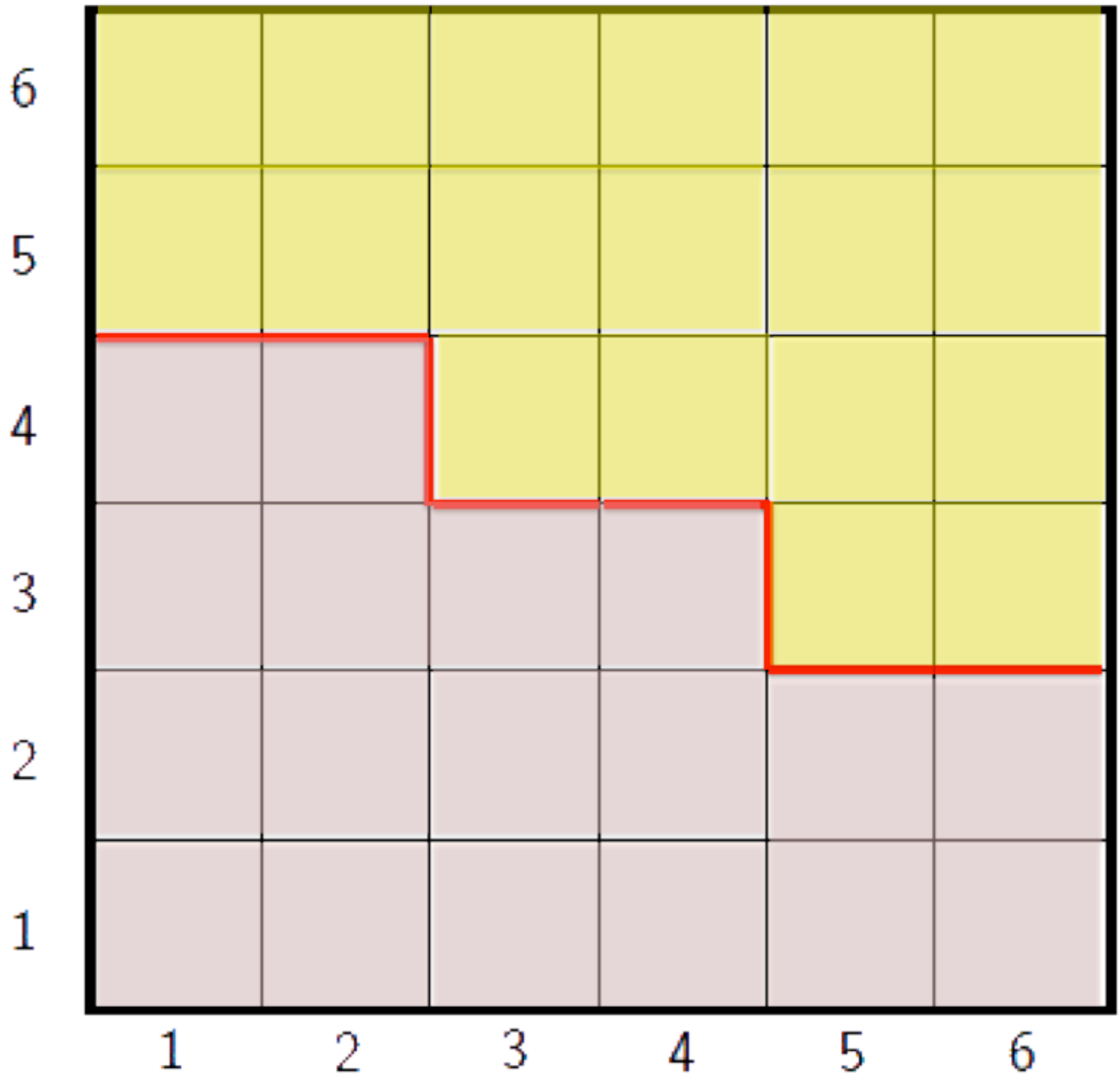

Figure S1 A and B. The board. The above are two examples of boards, where each player has an equal number of squares for placing their cowries on. The first one includes an image of two nearby colonies of *Pacifigorgia cairnsi* (bottom) and *Pacifigorgia* sp. (cf. *curta*) (top), illustrating the connection to actual scenarios. It is an idealized depiction of different colonies of the two species, where each colony is a square on the board. Figure S1 B shows a board of 6x6 columns and rows and divided in a more symmetrical manner. The numbers represent the position of the board, and when using dice of different colors, each can represent an axis.

A)

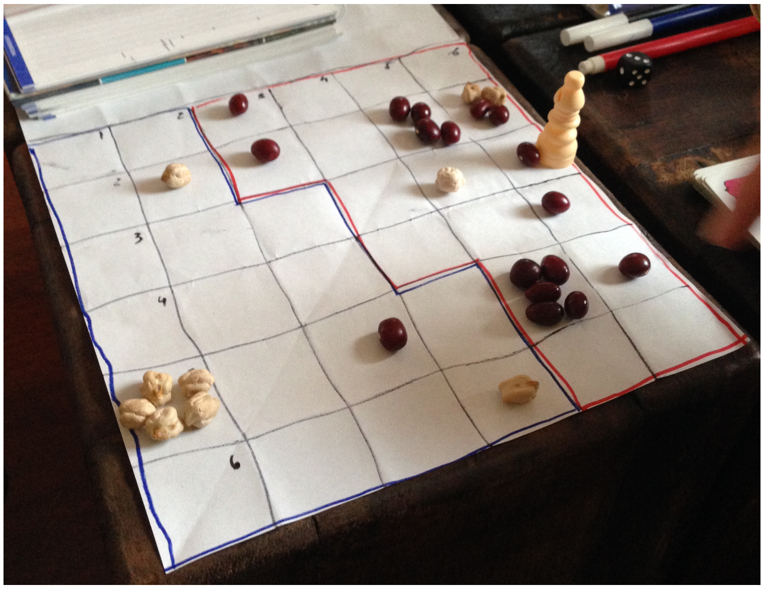

B)

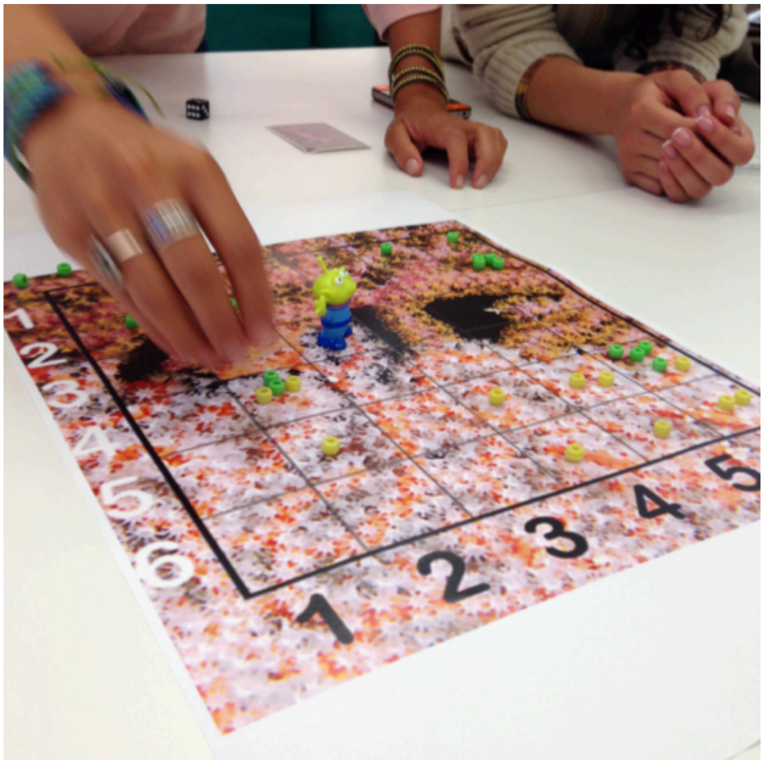

Figure S2 A and B Illustration of two implementations of the game. To play the game two kinds of colored cowry-like pieces are needed (e.g., beans and chickpeas as in A), and two different toy-like pieces to represent two kinds of predators (one is off the board, the super-predator). Players choose a side (a seafan population of specific color) and are given the same amount of "cowries" (6) to start with. The game begins with each player drawing cowries

randomly (with no control over their color) and ends when only one color remains (the winner) or when one seafan has been completely colonized by the cowries of matching color. We used the cards to assign these events as well as dice to specify the location if required (each dice for x or y axis of the board), except in the case of the predators, which had a physical presence on the board (the predator cannot move diagonally). The goal of the game is for each player to fill each and all of the squares of their color (Color 1 or color2) with at least one cowry of the color of their squares. There can be no more than six cowries in one particular square.

**Table S2**<br>**Adaption of the game for regular playing cards**
Picture Cards (including aces): are for use as movement cards (16 cards)<br>A player chooses one cowry to move to one adjacent square<br>Numbered cards:<br>- Spades are used as predator cards:<br>The number on the card shows how many steps the predator can take, eating all mismatched cowries in it´s path.<br>- Hearts are used as reproduction cards:<br>The number on the card representing the number of cowries to take part (and number over 6 will result in just six cowries congregating).<br>The dice are rolled to show position, and the closest cowries congregate there.<br>If all cowries are the same color the player with that color keeps the card.<br>- Clubs are used as recruitment cards:<br>The number on the card representing the number of cowries added,<br>the dice are rolled to show position, and cowries are placed there or in adjacent squares,<br>if more than six cowries are in one square the last ones added will die (be removed)was it the last ones added or did the player have control over which ones to remove?<br>If the player has a reproduction card they may play it and add cowries of their own colour, if not cowries are drawn randomly.<br>- Diamonds are used for the less probable events:<br>9 and 10 can be used for the super predator.<br>The dice are rolled to determine the position of attack and then the player chooses a corner of the square and the predator attacks all cowries adjacent to this (up to 4 squares)any cowries in these square are removed from the game board.<br>6,7 and 8 can be used as invasive species cards.<br>The dice are rolled to show a position, all cowries must move to adjacent squares, if there are more than 6 cowries in one square the most recent arrivals die (are removed).<br>2,3,4 and 5 can be used as Sea Fan disease cards<br>The dice are rolled and all cowries in that square are removed.

Table S2

Summarized playing rules of the Masquerade game

Each player should place six randomly chosen cowries on each their side of the board,
A predator on the center of the board and a super predator (outside the board waiting)
Each player then throws a die, and the player with the highest number gets to start; the die can be thrown again to decide the color that each player chooses, in case of disagreement.
In each turn a player:

- Moves one of his/her cowries by one space
- Draws a card (from the "events" cards) and follows the instructions of the card. These include:
- Reproduction
- Spawning
- Movement of the predator
- Catastrophic (also favorable) events such as pollution, disease, etc…
- Additional movement

The objective of the game is for each player to fill each and all of the squares of their color (Color 1 or color2) with at least cowry of the color of their squares.
There can be no more than six cowries in one particular square.

Make sure you read the paper before you play! Enjoy!

References

Long DE. 2012. The politics of teaching evolution, science education standards, and being a creationist. *Journal of Research in Science Teaching* 49:122–139.

Sadler TD., Romine WL., Stuart PE., Merle-Johnson D. 2013. Game-Based Curricula in Biology Classes: Differential Effects Among Varying Academic Levels. *Journal of Research in Science Teaching* 50:479–499.

Teaching and Student Services. 2008. Coral Reefs: Science and COnservation.

Yates TB., Marek EA. 2014. Teachers teaching misconceptions: a study of factors contributing to high school biology students' acquisition of biological evolution-related misconceptions. *Evolution: Education and Outreach* 7:7.

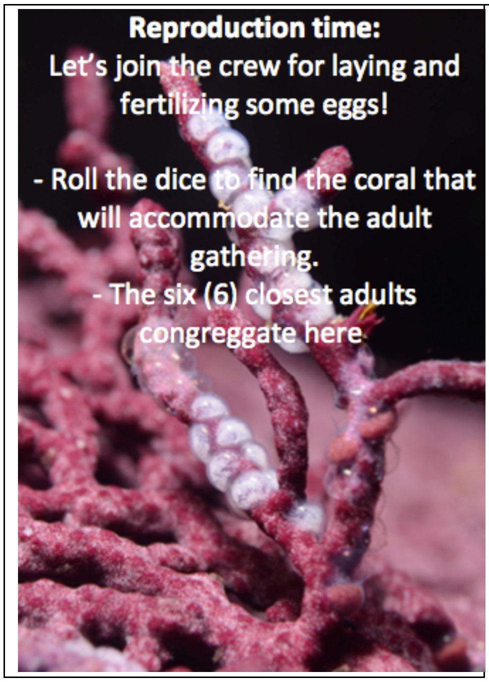
Reproduction time:
Let's join the crew for laying and fertilizing some eggs!
- Roll the dice to find the coral that will accommodate the adult gathering.
- The six (6) closest adults congreggate here
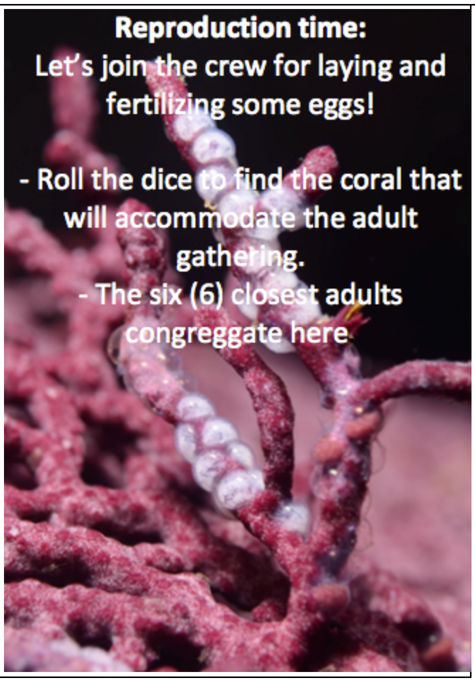
Reproduction time:
Let's join the crew for laying and fertilizing some eggs!
- Roll the dice to find the coral that will accommodate the adult gathering.
- The six (6) closest adults congreggate here
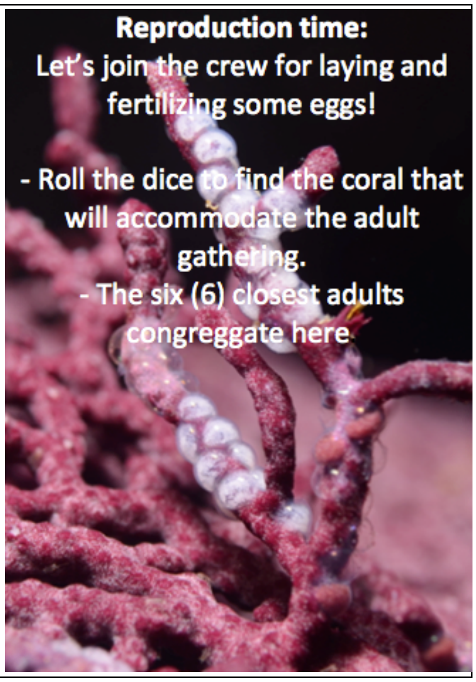
Reproduction time:
Let's join the crew for laying and fertilizing some eggs!
- Roll the dice to find the coral that will accommodate the adult gathering.
- The six (6) closest adults congreggate here
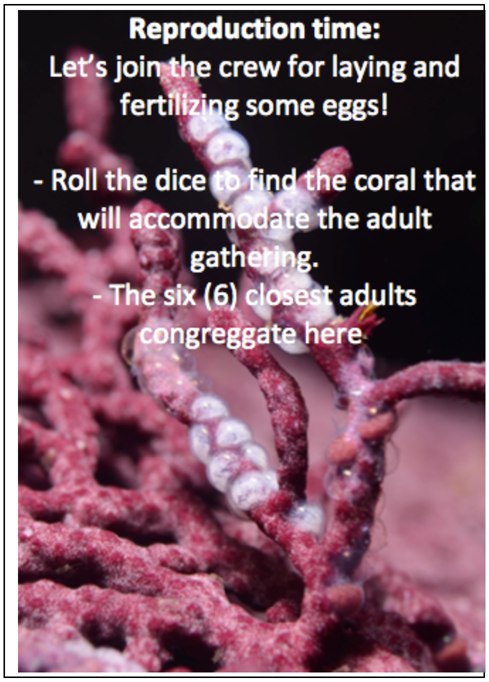
Reproduction time:
Let's join the crew for laying and fertilizing some eggs!
- Roll the dice to find the coral that will accommodate the adult gathering.
- The six (6) closest adults congreggate here
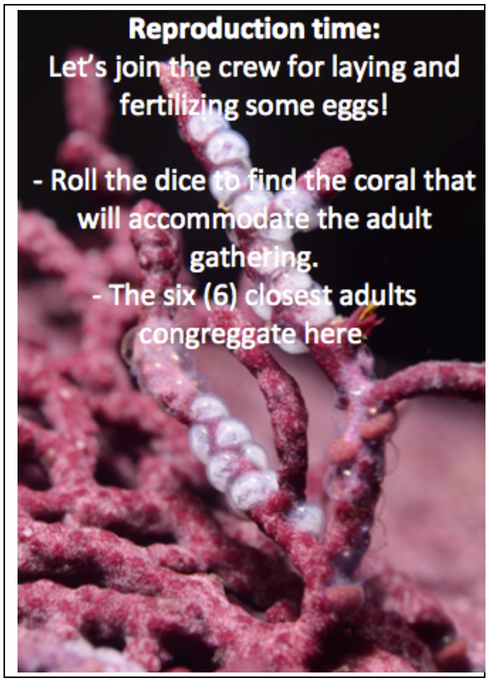
Reproduction time:
Let's join the crew for laying and fertilizing some eggs!
- Roll the dice to find the coral that will accommodate the adult gathering.
- The six (6) closest adults congreggate here
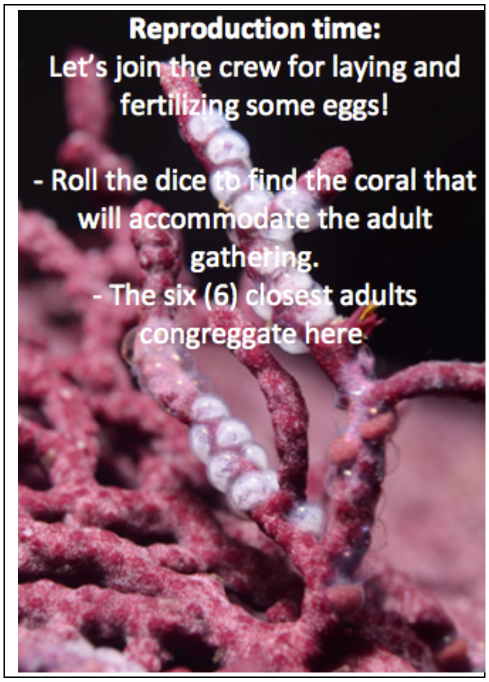
Reproduction time:
Let's join the crew for laying and fertilizing some eggs!
- Roll the dice to find the coral that will accommodate the adult gathering.
- The six (6) closest adults congreggate here
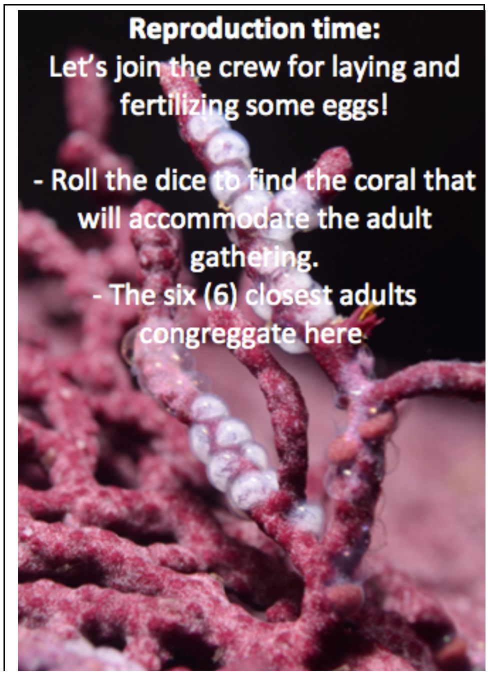
Reproduction time:
Let's join the crew for laying and fertilizing some eggs!
- Roll the dice to find the coral that will accommodate the adult gathering.
- The six (6) closest adults congreggate here
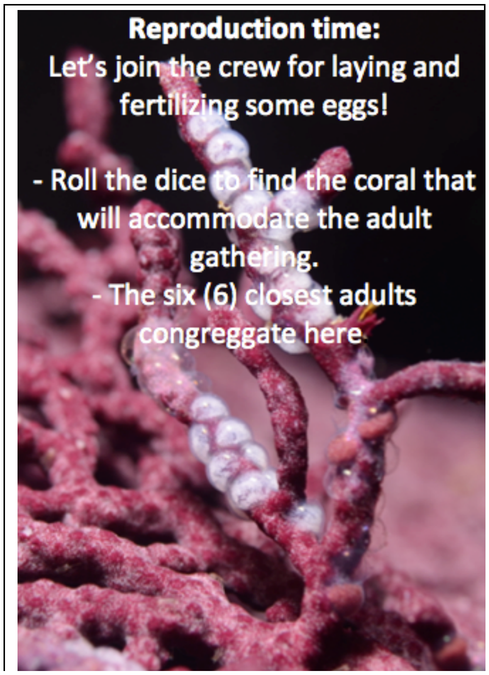
Reproduction time:
Let's join the crew for laying and fertilizing some eggs!
- Roll the dice to find the coral that will accommodate the adult gathering.
- The six (6) closest adults congreggate here
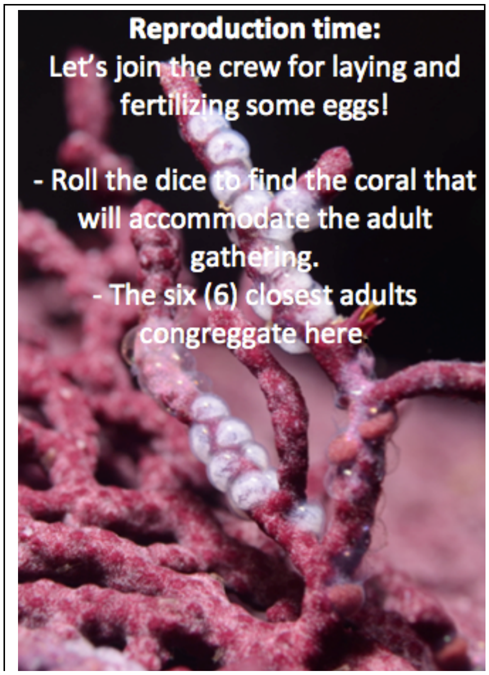
Reproduction time:
Let's join the crew for laying and fertilizing some eggs!
- Roll the dice to find the coral that will accommodate the adult gathering.
- The six (6) closest adults congreggate here

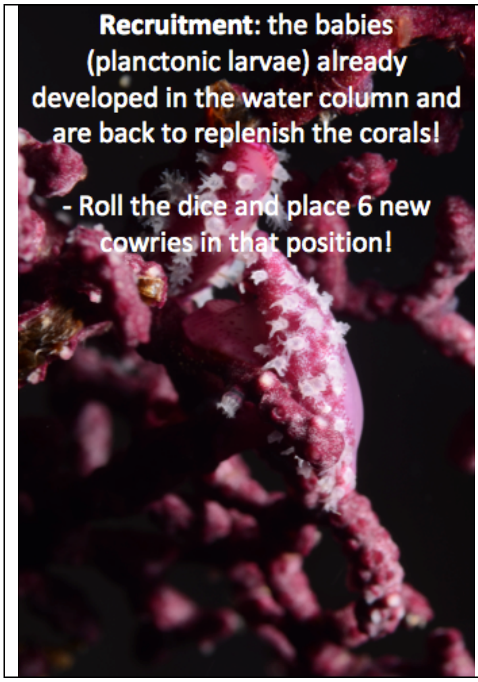
Recruitment: the babies (planctonic larvae) already developed in the water column and are back to replenish the corals!
- Roll the dice and place 6 new cowries in that position!

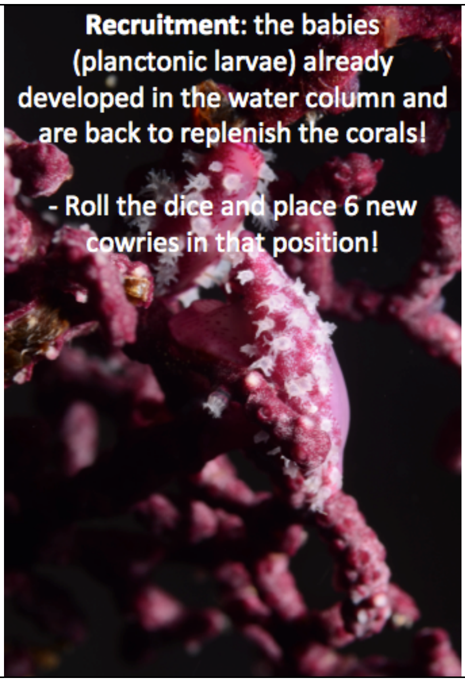
Recruitment: the babies (planctonic larvae) already developed in the water column and are back to replenish the corals!
- Roll the dice and place 6 new cowries in that position!

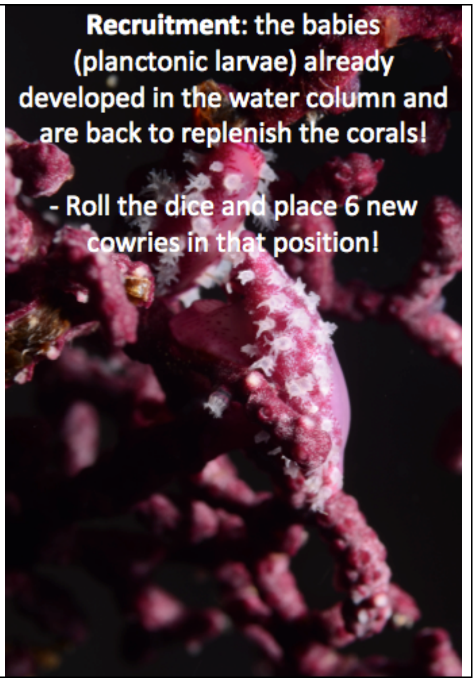
Recruitment: the babies (planctonic larvae) already developed in the water column and are back to replenish the corals!
- Roll the dice and place 6 new cowries in that position!

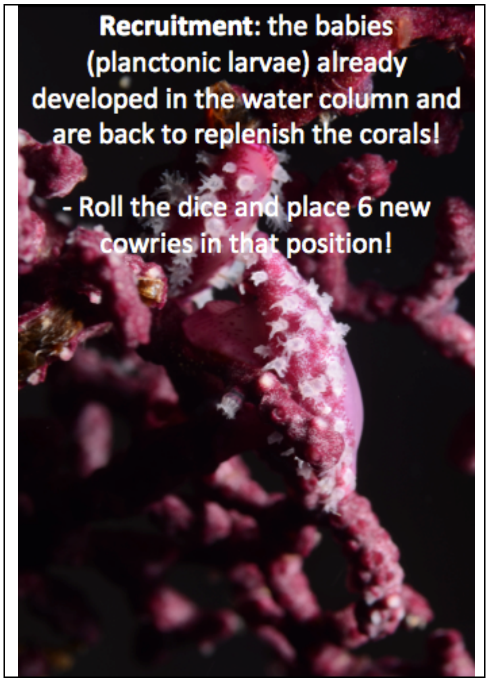
Recruitment: the babies (planctonic larvae) already developed in the water column and are back to replenish the corals!
- Roll the dice and place 6 new cowries in that position!

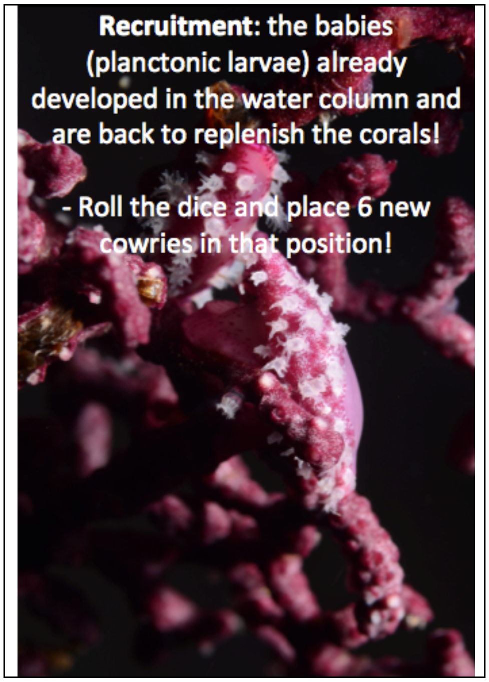
Recruitment: the babies (planctonic larvae) already developed in the water column and are back to replenish the corals!
- Roll the dice and place 6 new cowries in that position!

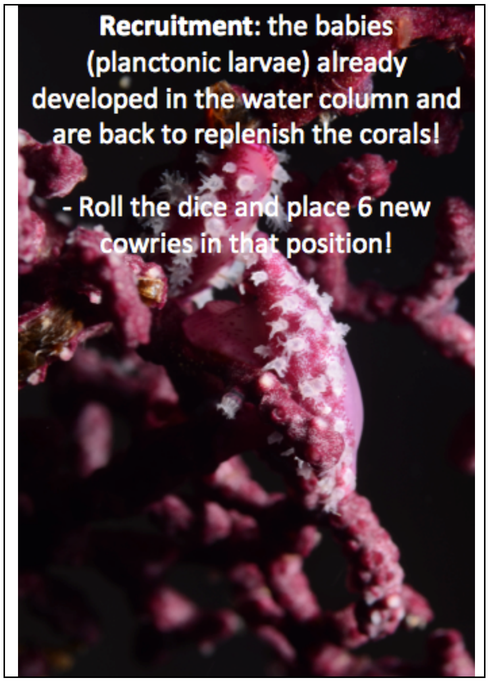
Recruitment: the babies (planctonic larvae) already developed in the water column and are back to replenish the corals!
- Roll the dice and place 6 new cowries in that position!

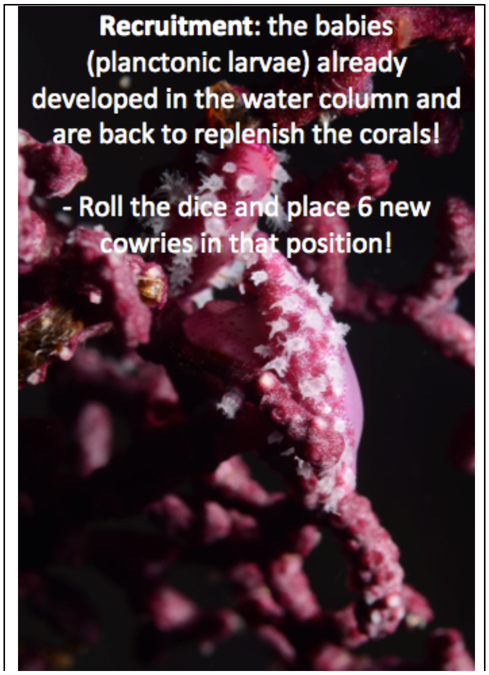
Recruitment: the babies (planctonic larvae) already developed in the water column and are back to replenish the corals!
- Roll the dice and place 6 new cowries in that position!

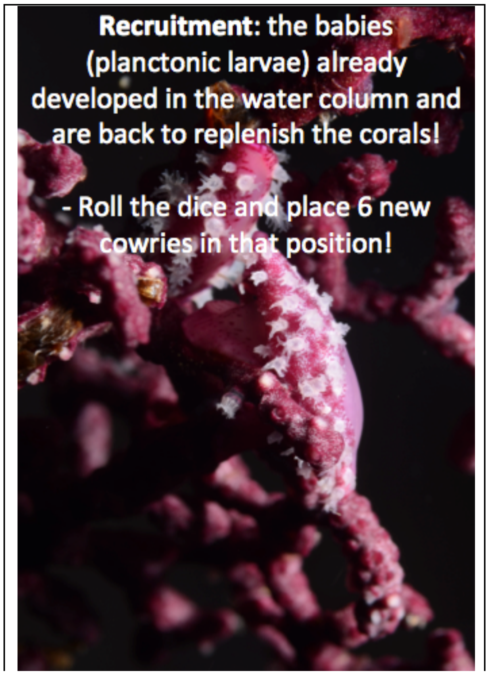
Recruitment: the babies (planctonic larvae) already developed in the water column and are back to replenish the corals!
- Roll the dice and place 6 new cowries in that position!

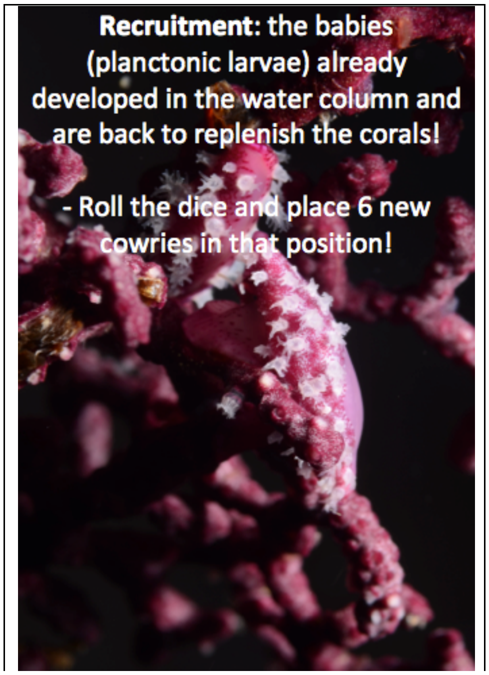
Recruitment: the babies (planctonic larvae) already developed in the water column and are back to replenish the corals!
- Roll the dice and place 6 new cowries in that position!

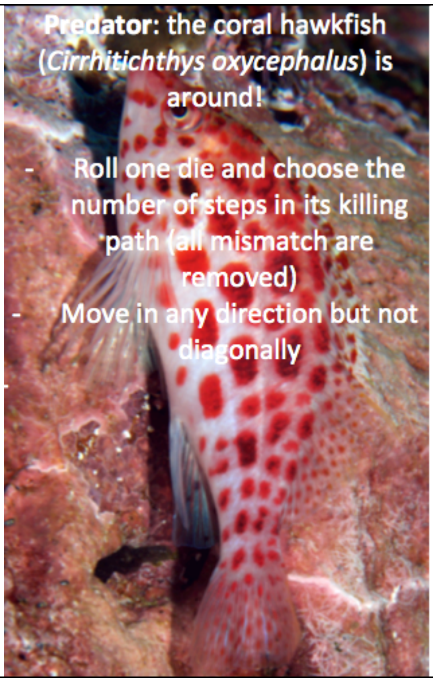
Predator: the coral hawkfish (Cirrhitichthys oxycephalus) is around!
- Roll one die and choose the number of steps in its killing path (all mismatch are removed)
- Move in any direction but not diagonally

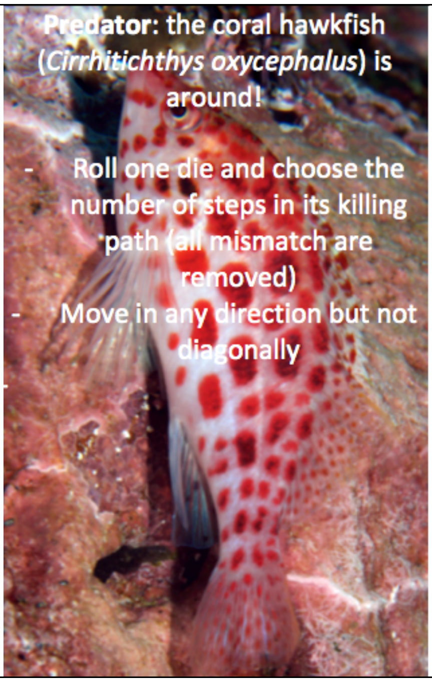
Predator: the coral hawkfish (Cirrhitichthys oxycephalus) is around!
- Roll one die and choose the number of steps in its killing path (all mismatch are removed)
- Move in any direction but not diagonally

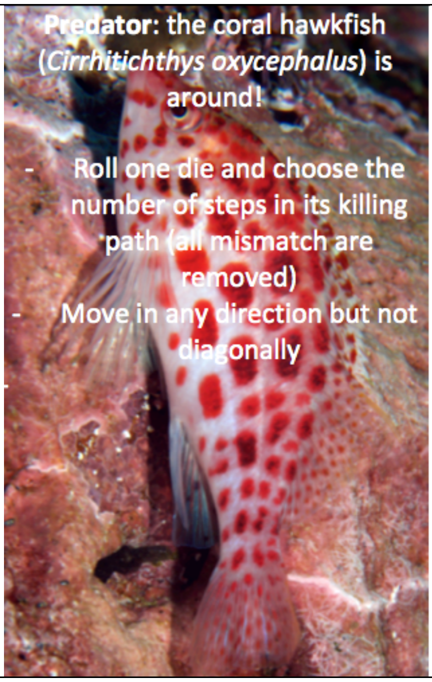
Predator: the coral hawkfish (Cirrhitichthys oxycephalus) is around!
- Roll one die and choose the number of steps in its killing path (all mismatch are removed)
- Move in any direction but not diagonally

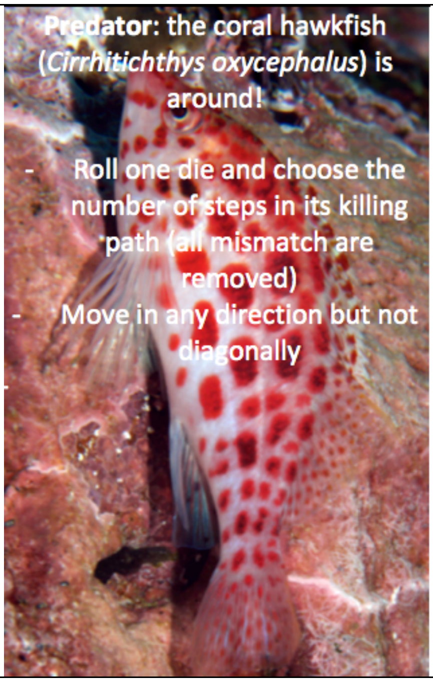
Predator: the coral hawkfish (Cirrhitichthys oxycephalus) is around!
- Roll one die and choose the number of steps in its killing path (all mismatch are removed)
- Move in any direction but not diagonally

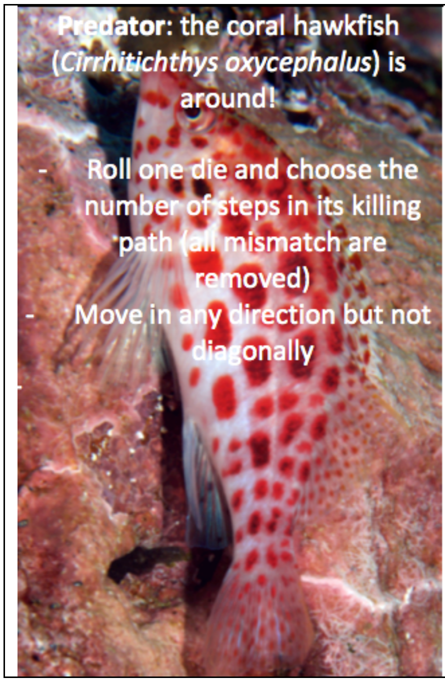
Predator: the coral hawkfish (Cirrhitichthys oxycephalus) is around!
- Roll one die and choose the number of steps in its killing path (all mismatch are removed)
- Move in any direction but not diagonally

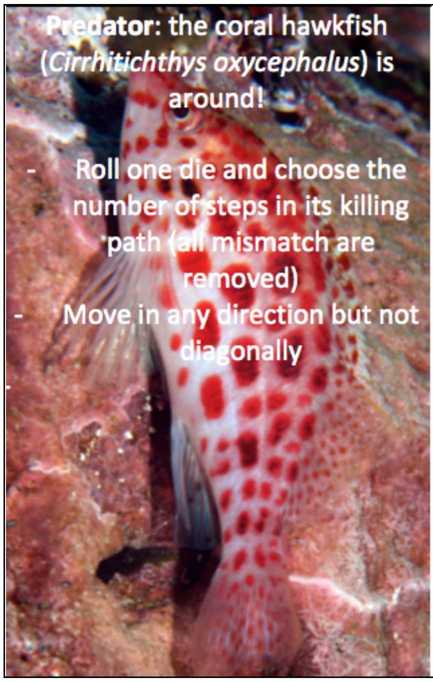
Predator: the coral hawkfish (Cirrhitichthys oxycephalus) is around!
- Roll one die and choose the number of steps in its killing path (all mismatch are removed)
- Move in any direction but not diagonally

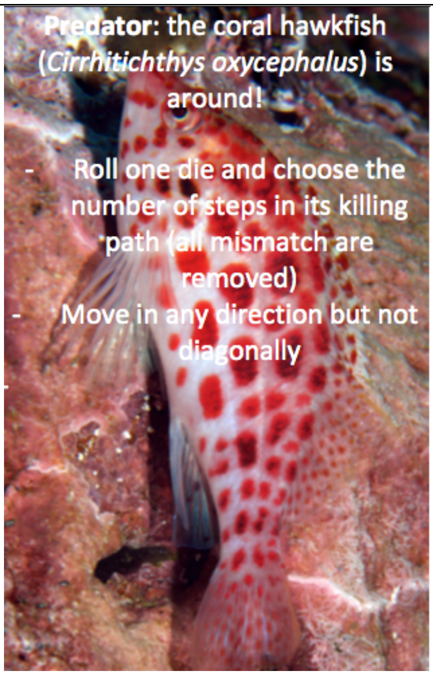
Predator: the coral hawkfish (Cirrhitichthys oxycephalus) is around!
- Roll one die and choose the number of steps in its killing path (all mismatch are removed)
- Move in any direction but not diagonally

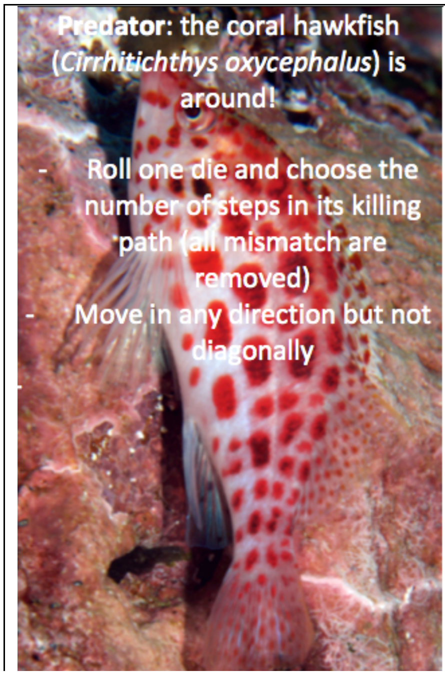
Predator: the coral hawkfish (Cirrhitichthys oxycephalus) is around!
- Roll one die and choose the number of steps in its killing path (all mismatch are removed)
- Move in any direction but not diagonally

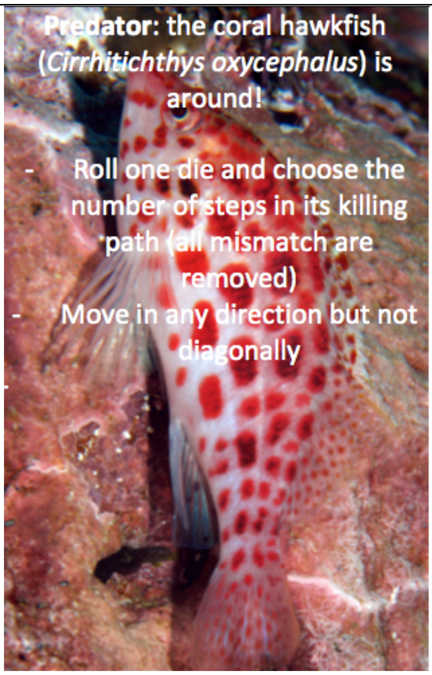
Predator: the coral hawkfish (Cirrhitichthys oxycephalus) is around!
- Roll one die and choose the number of steps in its killing path (all mismatch are removed)
- Move in any direction but not diagonally

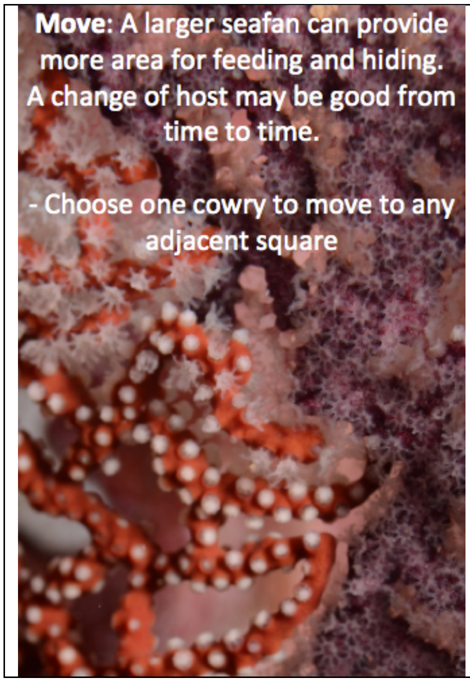
Move: A larger seafan can provide more area for feeding and hiding. A change of host may be good from time to time.
- Choose one cowry to move to any adjacent square

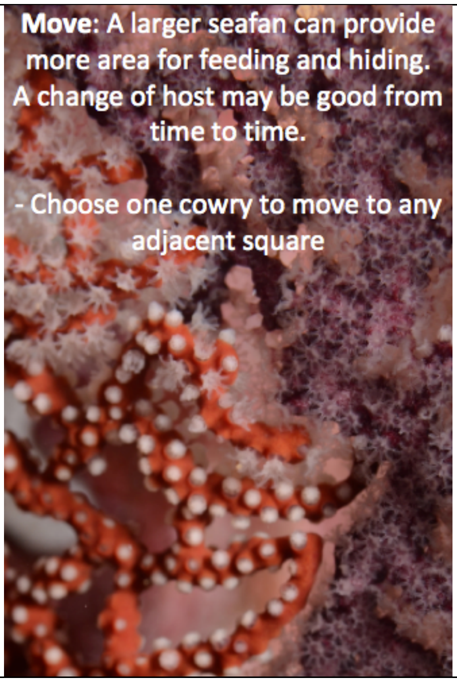
Move: A larger seafan can provide more area for feeding and hiding. A change of host may be good from time to time.
- Choose one cowry to move to any adjacent square

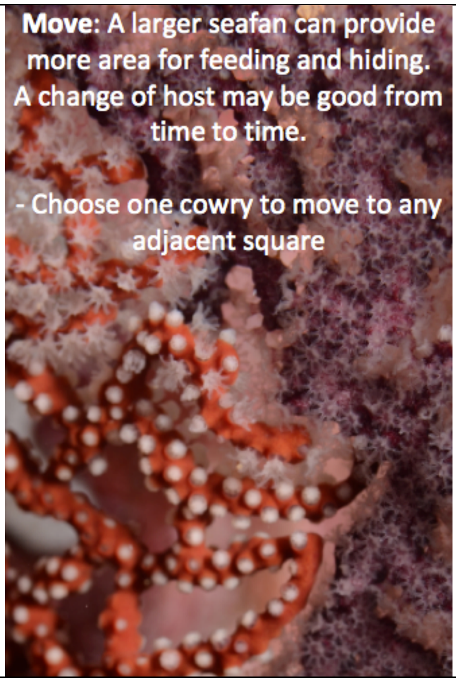
Move: A larger seafan can provide more area for feeding and hiding. A change of host may be good from time to time.
- Choose one cowry to move to any adjacent square

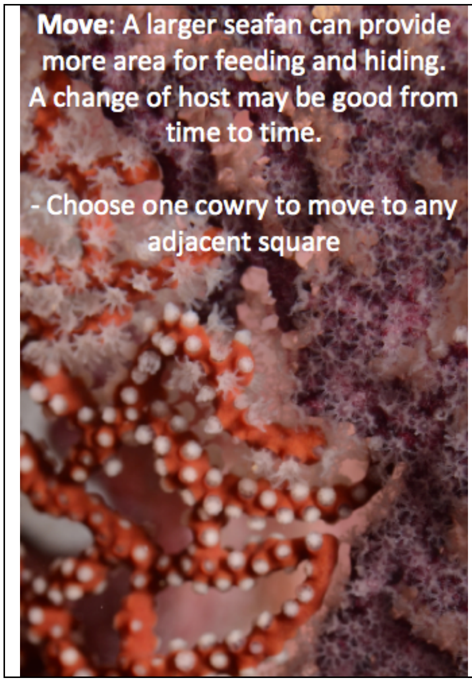
Move: A larger seafan can provide more area for feeding and hiding. A change of host may be good from time to time.
- Choose one cowry to move to any adjacent square

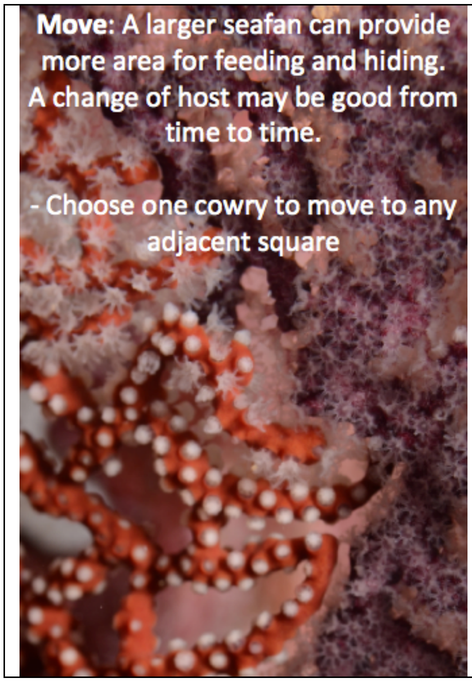
Move: A larger seafan can provide more area for feeding and hiding. A change of host may be good from time to time.
- Choose one cowry to move to any adjacent square

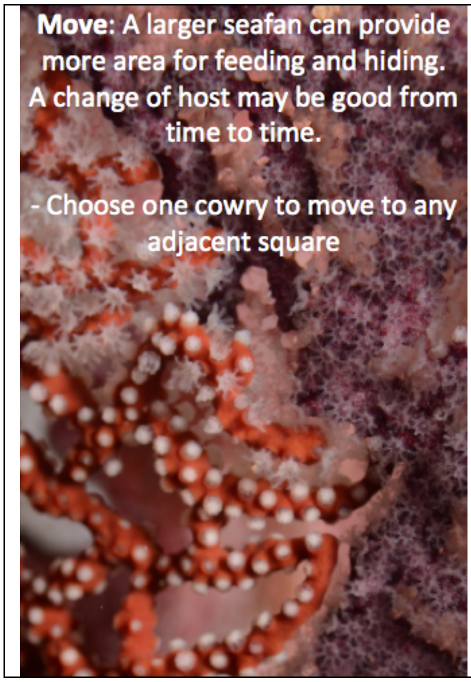
Move: A larger seafan can provide more area for feeding and hiding. A change of host may be good from time to time.
- Choose one cowry to move to any adjacent square

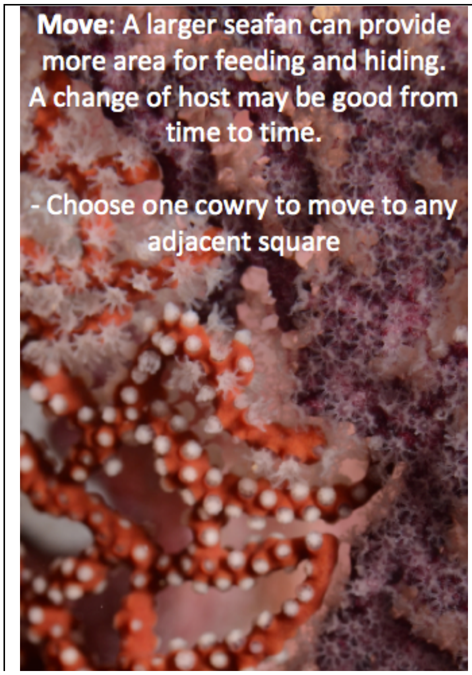
Move: A larger seafan can provide more area for feeding and hiding. A change of host may be good from time to time.
- Choose one cowry to move to any adjacent square

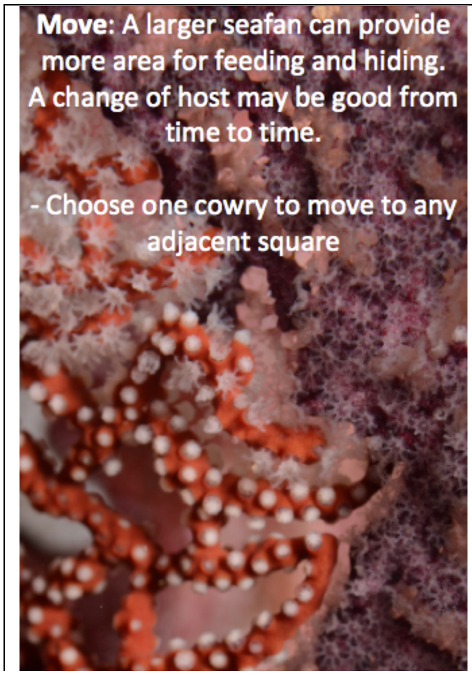
Move: A larger seafan can provide more area for feeding and hiding. A change of host may be good from time to time.
- Choose one cowry to move to any adjacent square

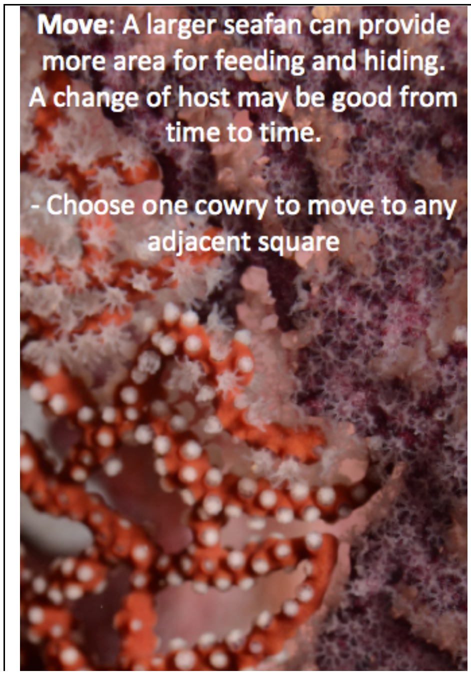
Move: A larger seafan can provide more area for feeding and hiding. A change of host may be good from time to time.
- Choose one cowry to move to any adjacent square

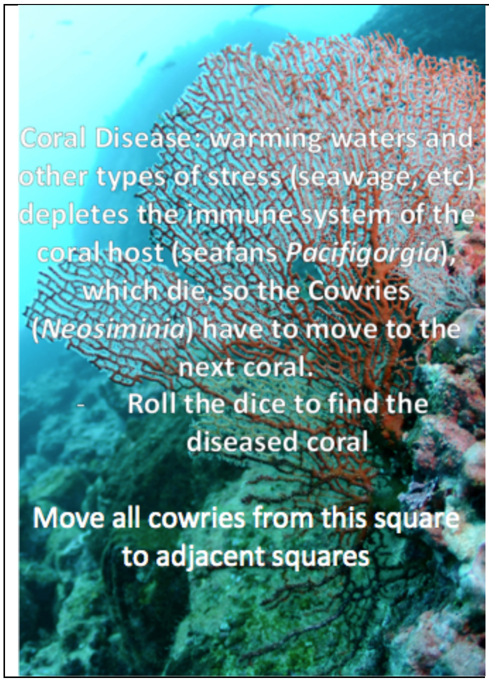

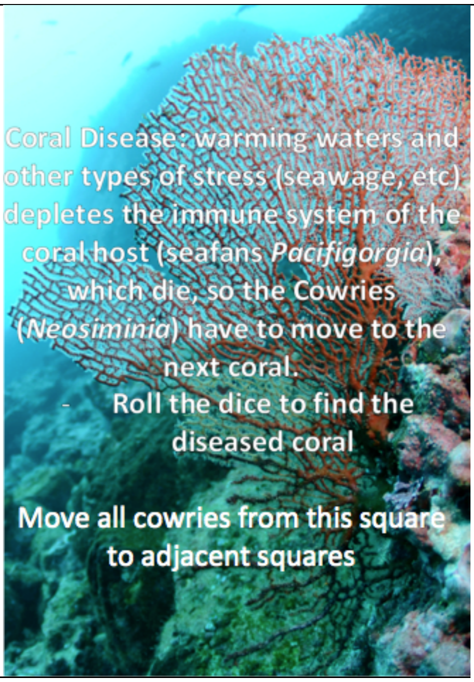

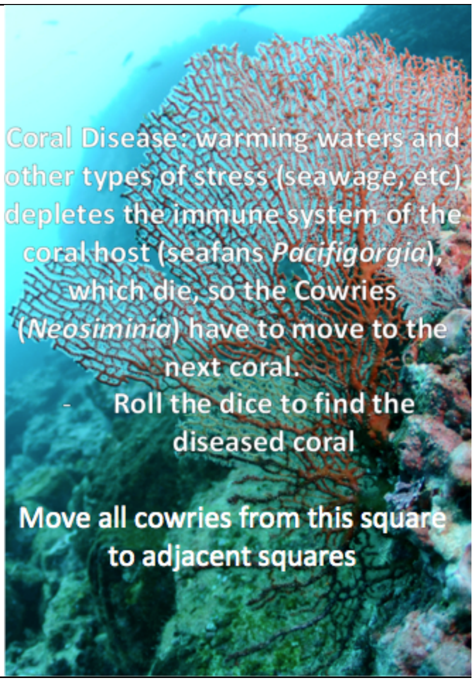

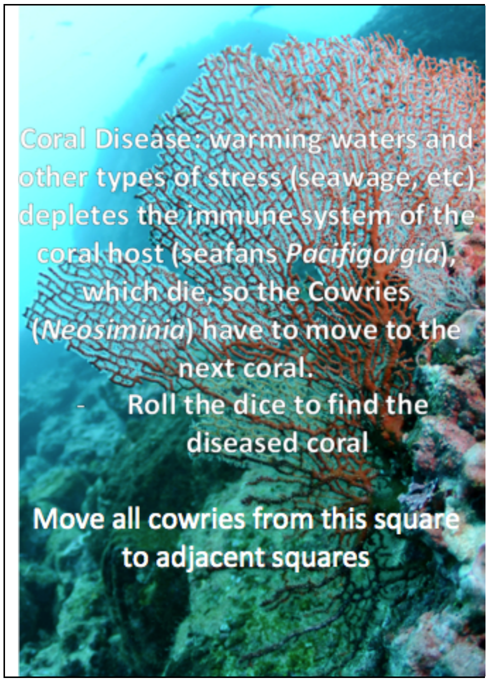

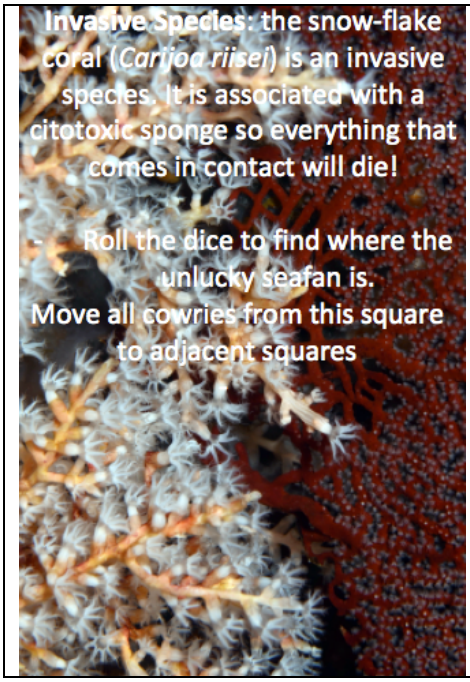

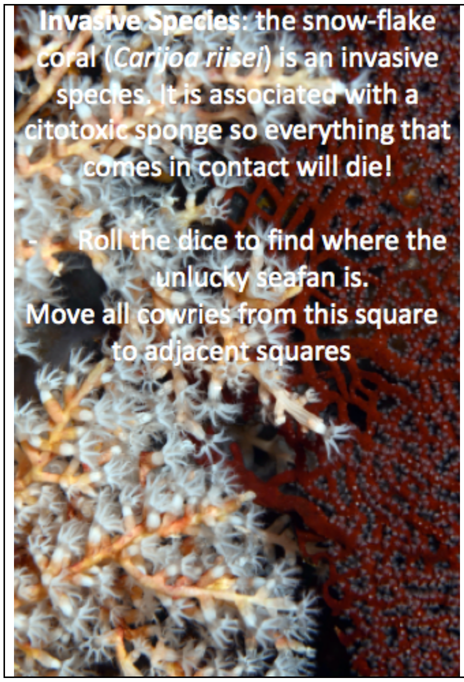

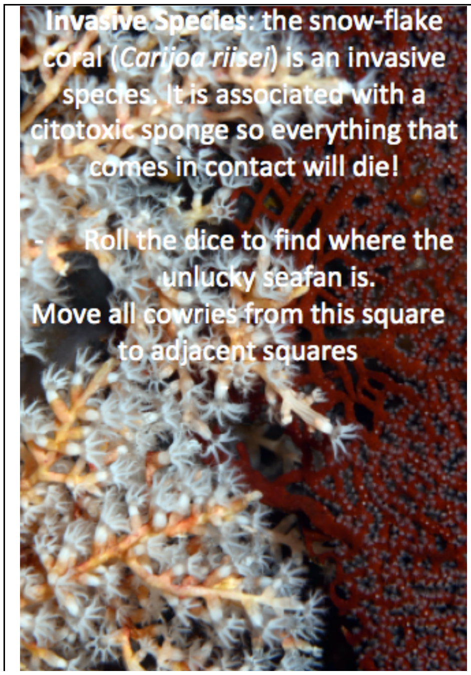

The Masquerade game: To play the game we need two kinds of colored cowry-like pieces (e.g., beans and chickpeas, see image above), and two different toy-like pieces for two kinds of predators. Players choose a side (a seafan populations of specific color) and are given the same amount of "cowries" (6) to start with. The game begins with each player drawing cowries randomly (with no control over which color) and ends when only one color remains (the winner) or when one seafan has been completely colonized by the cowries of matching color.

Events cards & dice: The cards are used to assign events in the population as well as dice to specify the location if required (each dice for x or y axis of the board), except in the case of the predators, which had a physical presence on the board (the predator can not move diagonally).

The goal of the game is for each player to fill each and all of the squares of their color (Color 1 or color2) with at least one cowry of the color of their squares. There can be no more than six cowries in one particular square. Enjoy!

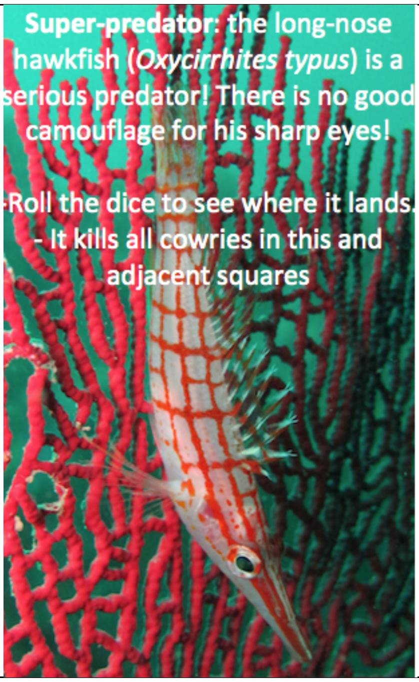
Super-predator: the long-nose hawkfish (*Oxycirrhites typus*) is a serious predator! There is no good camouflage for his sharp eyes!
Roll the dice to see where it lands.
- It kills all cowries in this and adjacent squares

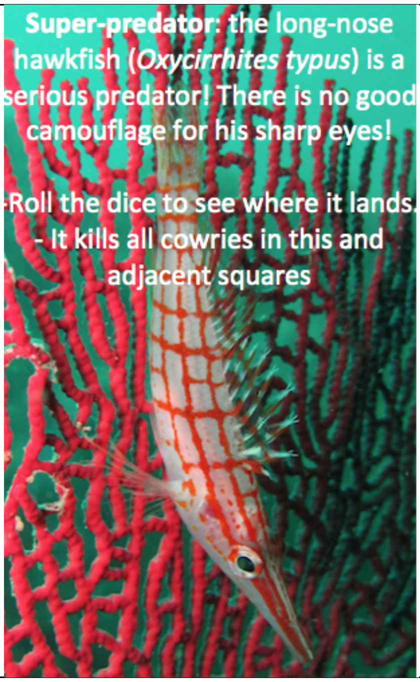
Super-predator: the long-nose hawkfish (*Oxycirrhites typus*) is a serious predator! There is no good camouflage for his sharp eyes!
Roll the dice to see where it lands.
- It kills all cowries in this and adjacent squares

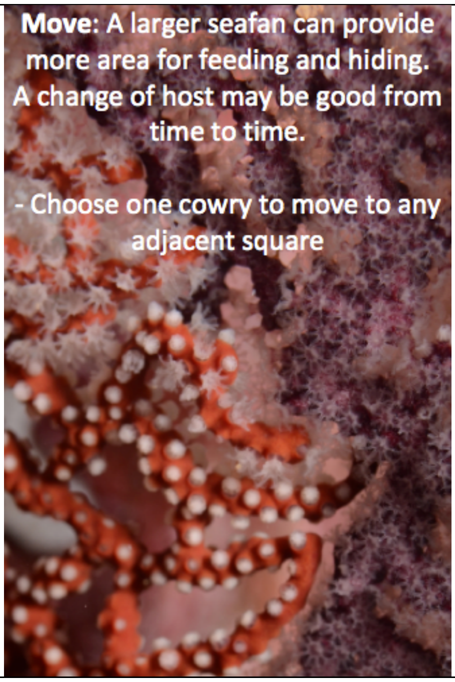
Move: A larger seafan can provide more area for feeding and hiding. A change of host may be good from time to time.
- Choose one cowry to move to any adjacent square

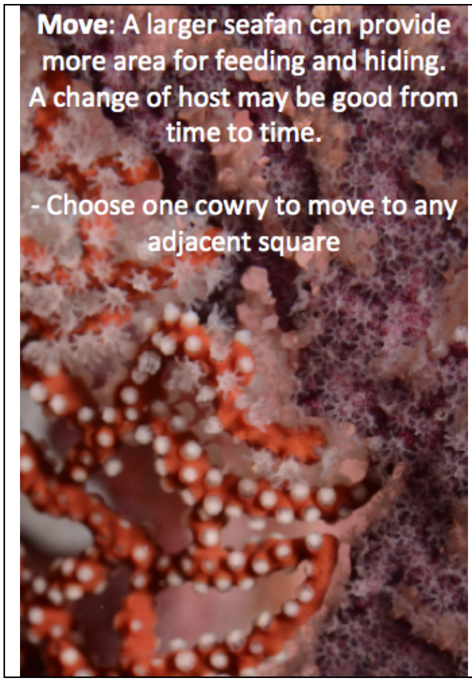
Move: A larger seafan can provide more area for feeding and hiding. A change of host may be good from time to time.
- Choose one cowry to move to any adjacent square

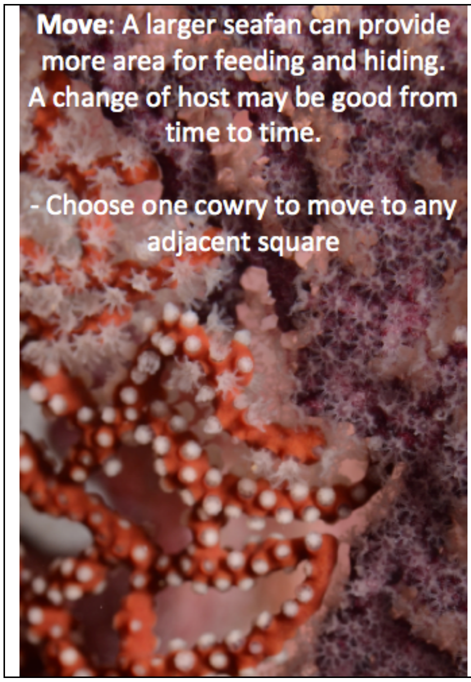
Move: A larger seafan can provide more area for feeding and hiding. A change of host may be good from time to time.
- Choose one cowry to move to any adjacent square

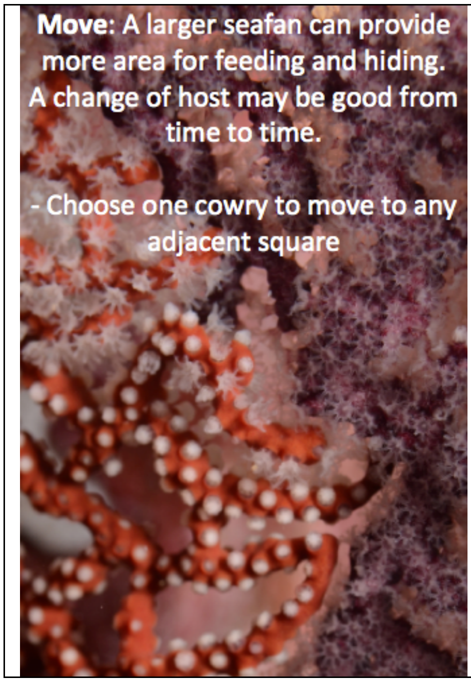
Move: A larger seafan can provide more area for feeding and hiding. A change of host may be good from time to time.
- Choose one cowry to move to any adjacent square

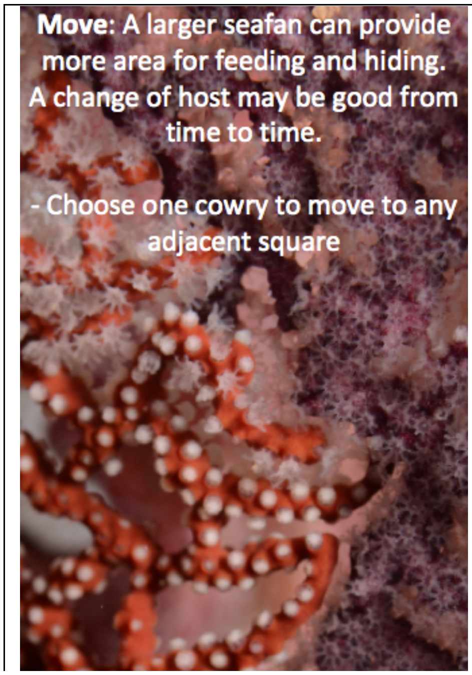
Move: A larger seafan can provide more area for feeding and hiding. A change of host may be good from time to time.
- Choose one cowry to move to any adjacent square

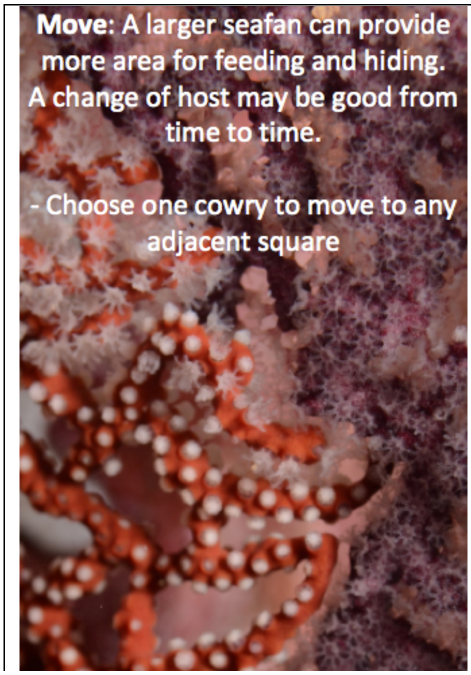
Move: A larger seafan can provide more area for feeding and hiding. A change of host may be good from time to time.
- Choose one cowry to move to any adjacent square

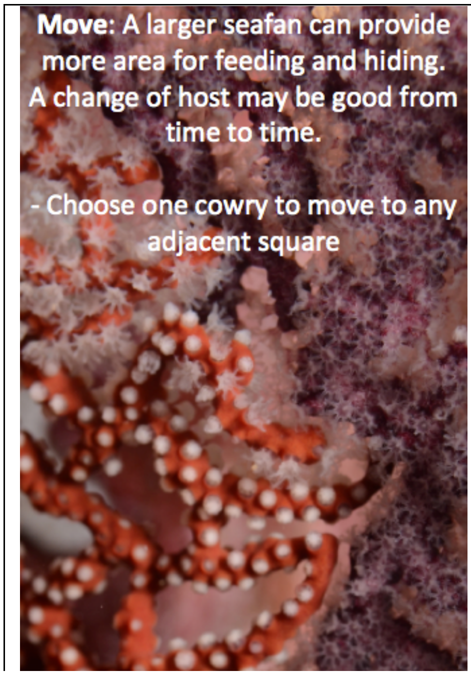
Move: A larger seafan can provide more area for feeding and hiding. A change of host may be good from time to time.
- Choose one cowry to move to any adjacent square

Masquerade

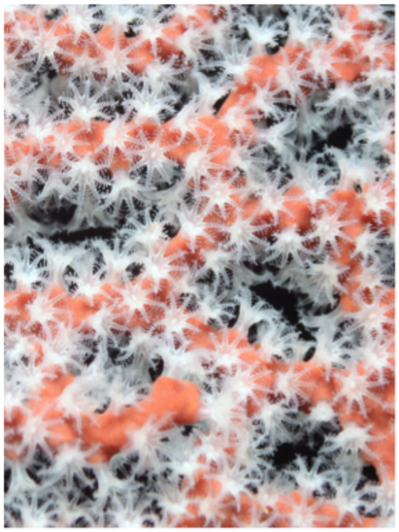

Masquerade

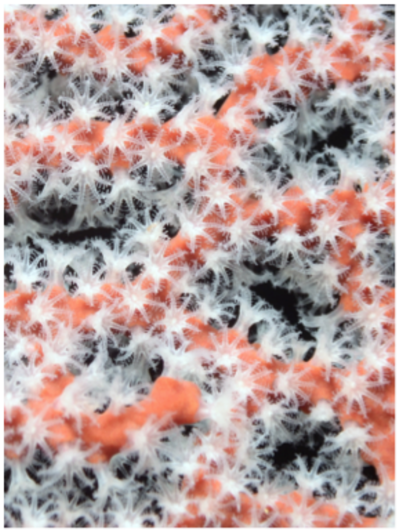

Masquerade

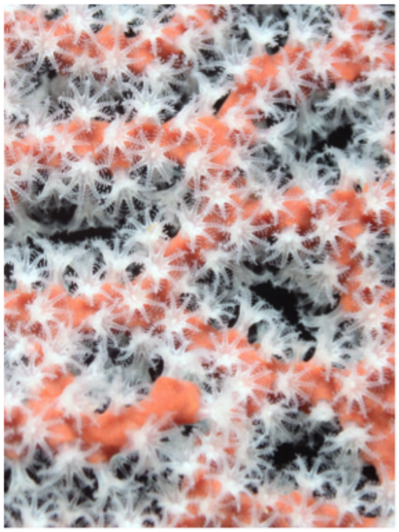

Masquerade

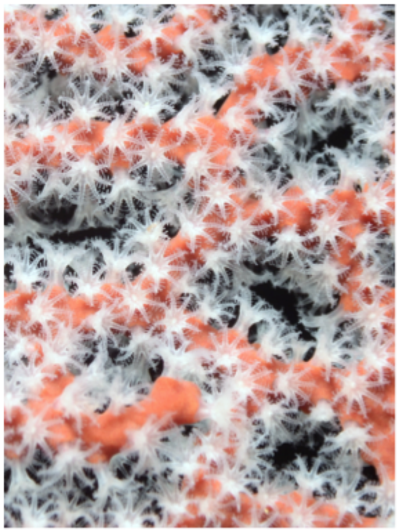

Masquerade

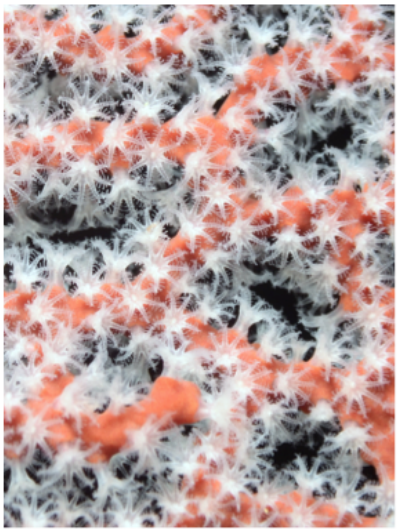

Masquerade

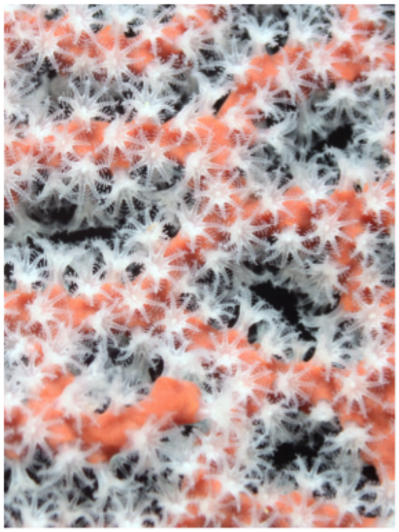

Masquerade

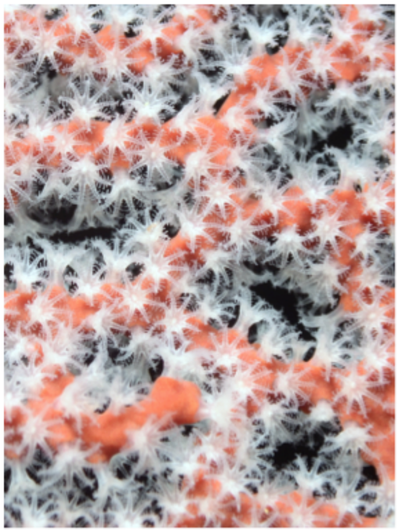

Masquerade

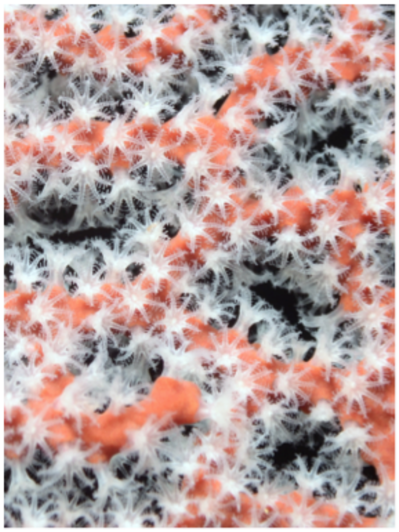

Masquerade

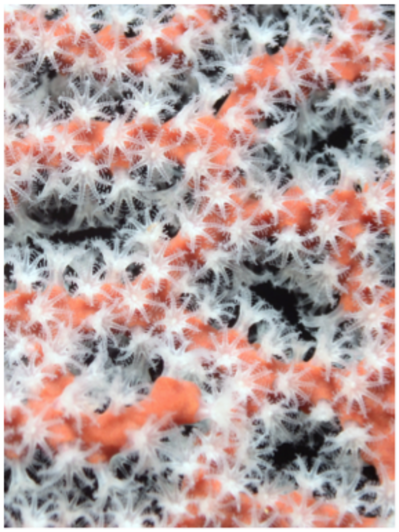

Supplement: Supplemental Information 1 — Development of a board game for teaching, playing and outreach (includes tables, figures and a game kit). [file peerj-04-2051-s001.pdf]
